# Surface Quality Evaluation of Removable Thermoplastic Dental Appliances Related to Staining Beverages and Cleaning Agents

**DOI:** 10.3390/polym12081736

**Published:** 2020-08-03

**Authors:** Liliana Porojan, Roxana-Diana Vasiliu, Sorin-Daniel Porojan, Mihaela-Ionela Bîrdeanu

**Affiliations:** 1 Department of Dental Prostheses Technology (Dental Technology), “Victor Babeș” University of Medicine and Pharmacy Timișoara, Romania, Eftimie Murgu Sq. no. 2, 300041 Timișoara, Romania; sliliana@umft.ro; 2 Department of Oral Rehabilitation (Dental Technology), “Victor Babeș” University of Medicine and Pharmacy Timișoara, Romania, Eftimie Murgu Sq. no. 2, 300041 Timișoara, Romania; porojan.sorin@umft.ro; 3 National Institute for Research and Development in Electrochemistry and Condensed Matter, 300569 Timisoara, Romania; mihaelabirdeanu@gmail.com

**Keywords:** thermoplastic materials, color stability, surface topography, staining beverage, cleaning method

## Abstract

(1) Background: Thermoplastic materials are not inert and subject to changes in the oral environment, which affect their surface quality. Color stability and topographic characteristics of clear thermoplastic appliances are critical considerations. The study aimed to evaluate the optical changes and surface topography of different thermoplastic materials related to staining beverages and cleaning agents. (2) Methods: Thermoplastic polyethylene terephthalate glycol (PET-G) material specimens were selected for the study: S (Duran, Scheu-Dental GmbH, Iserlohn, Germany), D (Biolon, Dreve Dentamid GmbH, Unna, Germany), and B (Crystal, Bio Art Dental Equipment, Sao Carlos, Brazil). Four different media were involved for immersion: coffee (C) and black tea (T) at 55 °C, Coca-Cola (K) at 5 °C, and distilled water (W) at 22 °C. As for cleaning, chemical options and mechanical brushing were selected (P-powder, T-tablets, and X-brushing). Color changes, and mean surface roughness were measured at 24 h, 48 h, and after 7 days. Statistical analysis was performed. After the testing period, atomic force microscopy (AFM) analyses and SEM images were registered in order to characterize the surface topography. (3) Results: Quantitative color change evaluations revealed a slight change in color after 24 h and an extremely marked change after 48 h, respective 7 days. Mean roughness values are kept below the clinically acceptable limit of 0.20 µm for all samples. Related to mean nanoroughness values Sa, and 3D evaluations of the surface quality, Biolon samples have demonstrated the most constant behavior, while Crystal samples are visibly influenced by water immersion. Related to the cleaning method, the topography of Duran samples was influenced by mechanical brushing. (4) Conclusions: Nanoscale investigations provided high accuracy and more realistic surface quality examinations of the examined samples compared to profilometry. Both SEM and AFM should be used for a more detailed description of the surface topography.

## 1. Introduction

Thermoplastic materials have been introduced in dentistry for different removable appliances, for example, orthodontic retainers, aligners, prosthetic splints, night guards, and different trays [1]. This wide range of applications is linked to their good mechanical properties, biocompatibility, chemical stability, excellent esthetic characteristics, good formability, and low cost [2]. Thermoplastic materials are not inert and are sensitive to the changes in the oral environment, during contact with saliva, due to temperature variations, colored beverages, humidity, and different mechanical loadings. In vitro testing methods are unable to reproduce the clinical behavior, but it is important to take into consideration the water absorption, which has a mechanical effect related to the hygroscopic expansion [3,4].

Thermoplastic materials drew attention over time due to their aesthetics and through their application of thermoforming [5].

The majority of current removable appliances are modified polyethylene terephthalate glycol (PET-G), although polypropylene, polycarbonate, polyurethanes, copolyester, and many other materials are also used [6,7,8]. Structurally, PET-G materials are amorphous. They are clear because visible light can pass through these polymers. The degree of crystallization after thermoforming of an amorphous polymer is almost negligible [4].

The forming process includes a heating cycle, associated with vacuum or pressure forming, which could cause changes in their morphological and mechanical properties. Furthermore, their insertion in the oral environment submits them to other thermal, mechanical, and chemical degradation, changes that can be observed, including color changes and surface alterations [4,8].

Translucency and color stability of clear thermoplastic appliances are essential considerations for both patients and clinicians. However, the aesthetic properties of dental appliances are often influenced by various environmental factors, like humidity, temperature variations, and staining beverages [9]. Part of them could be avoided because it is recommended that all removable appliances be removed before eating and drinking, but studies have reported that the compliance of the patients is insufficient [10].

Maintaining the optical properties of removable clear appliances is a key concern. Mechanical and chemical cleaning methods are currently the two main cleaning methods available. Proper cleaning methods are important to maintain the integrity of the thermoplastic material [11,12]. The novelty of this research is brought by the materials and by the correlation between the optical and surface changes that occur after beverage immersion for an amount of time. This has great relevance in observing the behavior from a colorimetric and a structural perspective. Not all patients that wear these appliances have compliance with cleaning them properly and with avoiding coloring agents; this is why these studies’ purpose is to evaluate the optical and surface topographical behavior of these materials in simulated clinical conditions related to staining beverages and cleaning agents.

Studies related to these kinds of materials are usually carried out on the macroscopic scale, and the surface deterioration of PET-G removable appliances on the nanoscale is still an open question [13].

## 2. Materials and Methods

### 2.1. Specimen Preparation

Thermoplastic PET-G material specimens were selected for the study: Duran^®^ (Scheu-Dental GmbH, Iserlohn, Germany), Biolon^®^ (Dreve Dentamid GmbH, Unna, Germany), and Crystal ^®^ (Bio Art Dental Equipment, Sao Carlos, Brazil), with 1.0 mm thickness. The composition of the selected materials is the same. The materials were chosen with the same composition from different producers to compare their structural behavior. 

These selected materials indicate orthodontic use and were prepared accordingly. The materials are polyethylene terephthalate glycol (PET-G) [14]. PET-G is a non-crystallized amorphous polymer that has a higher glass transition temperature and water absorption rate compared to semicrystalline polymers. PET-G is a co-polymer of PET composed of 1, 4-cyclohexane two methanol (CHDM), ethylene glycol (EG), and terephthalic acid (TPA) [5,6].

The sheets were thermoformed, and specimens of 12 × 12 × 1 mm (*n* = 36) were randomly divided into four groups, according to the coloring media. 

Thermoforming was made using a pressure molding unit MINISTAR S^®^ (Scheu-Dental GmbH, Iserlohn, Germany). The specific parameters used for the thermoforming processes of each material are reported in Table 1. From each material, a sheet was preserved both before and after thermoforming. According to this, the total number of investigated samples was *n* = 42.

**Figure 1:**
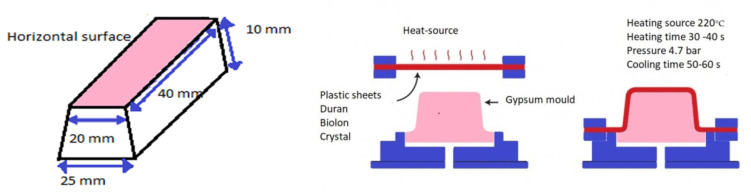
Fabrication of specimens to assess the color change and surface roughness, the gypsum mold, and the thermoforming process.

A gypsum mold was prepared and placed in the thermoforming machine. Heat and vacuum were applied during the process according to each manufacturer. The models obtained after thermoforming were removed, and the horizontal surface was used. Specimens were cut using scissors to avoid heat and deformations. The experimental part is presented in Figure 1.

Four different media were involved in immersion. A volume of 50 mL was taken from the prepared beverages for each immersion. The instant coffee solution consisted of 1.8 g of instant coffee powder (Jacobs Krönung, Jacobs Douwe Egberts, Amsterdam, The Netherlands) per 50 mL of boiling water. Black tea (Lipton Yellow Label, Unilever, London, UK), 1 bag per 200 mL of boiling water was used (steeped for 2 min). Coffee and tea were maintained at 55 °C in a thermostat. The Coca-Cola (Coca-Cola Company, Atlanta, GA, USA) coloring media was used as supplied and maintained at 5 °C in a thermostat. Distilled water was employed at 22 °C. The beverages were refreshed every 24 h. These temperatures were selected to simulate the oral environment. After that, each group of 9 samples per solution was split again into three groups of 3 shells, which were cleaned by one of the three chosen methods. From each group, one material was selected for each cleaning procedure. As for cleaning solutions, two types were chosen, chemical (powder and tablets) and mechanical (brushing).

Chemical cleaning procedure was chosen among various products from the market, Centron Cleaning Powder (Scheu, Iserlohn, Germany) and Corega Cleanser Tablets (Stafford-Miller, Dungarvan, Ireland). For Centron, a bag of 15 g was dissolved in 150 mL water, and the samples were immersed for 30 min. Corega tablets were dissolved in warm water, and samples were cleaned for 3 min. The mechanical cleaning procedure tooth brushing method, in which specimens were brushed with an electric toothbrushing machine from Philips Sonicare (Philips, Amsterdam, The Netherlands), with Colgate Total Original toothpaste, for 2 min, and rinsed in a water bath. The samples were cleaned using proper solutions or brushed every 24 h, over 7 days.

Before each investigation, all samples were washed with distilled water and dried. Abbreviations are listed in Table 2.

### 2.2. Color Change Evaluation

The color changes (ΔE*) were calculated based on the CIE L*a*b*color system. L* represents lightness (+ bright, and − dark), a* represents the color scale from red (+) to green (−), and b* the color scale from yellow (+) to blue (−).

The total color change value (ΔΕ*) was calculated according to Equation (1)

ΔΕ* = [(ΔL*)2 + (Δa*)2 +(Δb*)2]1/2,(1)

which represents the color difference before and after immersion.

Measurements were made before thermoforming, after thermoforming and before immersion, and after periods of 24 h, 48 h, and 7 days. 

The national bureau of standards (NBS) system was used to quantify the levels of color change (Table 3). To relate the color change to a clinical standard, the ΔE* values were converted into NBS units: NBS = ΔE* × 0.92 [10,15,16,17,18]. 

### 2.3. Surface Roughness Measurements

Surface roughness was quantified, using a profilometer Surftest SJ-201 (Mitutoyo, Kawasaki, Japan), with a contact stylus of 2 µm. Arithmetic average roughness (Ra) values were registered in 3 different directions; data were recorded, and mean values of the three measurements was calculated. The sampling length was 0.8 mm, and a force of 0.7 mN was applied. All registrations were recorded on dry surfaces, before thermoforming, after thermoforming, and after immersion of the samples in the corresponding beverages for 24 h, 48 h, and 7 days, related to the different cleaning methods.

### 2.4. Statistical Analysis

Analyse-it software (Analyse-it Software, Ltd., Leeds, UK) was chosen for the statistical analysis. Differences among the variables were made, and the unpaired t-test was chosen to evaluate the comparisons between the means. A *p*-value of under 0.05 was considered statistically significant. Spearman correlation was used to assess relationships between microroughness and color change. The strength of association between variables was related to 0–0.19 “very weak,” 0.20–0.39 “weak,” 0.40–0.59 “moderate,” 0.60–0.79 “strong,” 0.80–1.0 “very strong,” and the direction of the relationship (+ for the same direction, and − for the opposed direction).

### 2.5. Nanosurface Topographic Characterization by Atomic Force Microscopy (AFM) 

Each sample was examined before thermoforming, after thermoforming, and after the period of 7 days with an atomic force microscope Nanosurf Easy Scan 2 Advanced Research (NanosurfAG, Liestal, Switzerland), and values for average nanoroughness Sa (nm), peak height values Sp (nm), valley depth values Sv (nm) were registered. Atomic force microscopy (AFM) generated a three-dimensional image of the sample surface (4.52 µm × 4.52 µm) and nanoroughness measurements. 

### 2.6. Microstructure Analysis by Scanning Electron Microscopy (SEM)

The thermoplastic samples have been investigated for microstructure analysis, before thermoforming, after thermoforming, and after a7 days. Specimens from each group were subjected to SEM examination (San Francisco Estuary Institute, Richmond, CA, USA). SEM images were used for qualitative analysis to evaluate the microstructure of the materials. 

## 3. Results

### 3.1. Color Evaluation

After thermoforming, a slight change in color was registered (ΔE* = 0.63 for S, 0.77 for D, and 1.25 for B). Resulted in quantitative color change evaluations revealed a slight change in color after 24 h (ΔE* between 0.5 and 1.5) and an extremely marked change after 48 h, respective 7 days (ΔE* between 6 and 12). 

Related to the beverages after 24 h, color changes are in this descending order: C > K > W > T, with significant differences between tea and all other beverages. After 48 h, the changes decreased in this order: C > W > K > T, with significant differences between C and all other beverages, and between T and W. 

After 7 days, like after 24 h, the staining occurs in this order: C > K > W > T, with significant differences between coffee and tea, respective water, and between K and W.).

**Figure 2:**
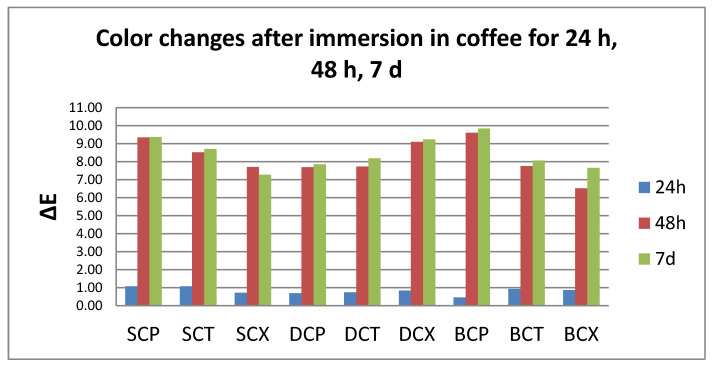
Mean values for ∆E parameter for all the tested values after immersion in coffee.

**Figure 3:**
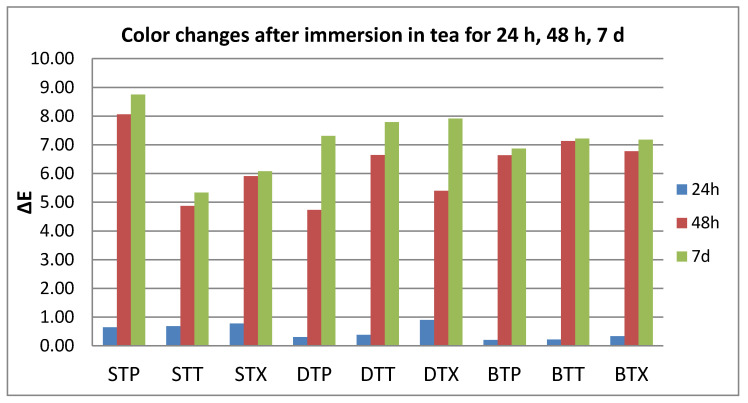
Mean values for ∆E parameter for all the tested values after immersion in tea.

**Figure 4:**
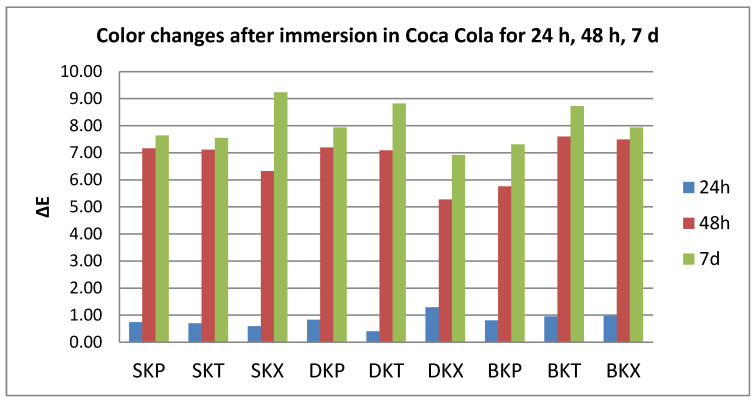
Mean values for ∆E parameter for all the tested values after immersion in Coca-Cola.

**Figure 5:**
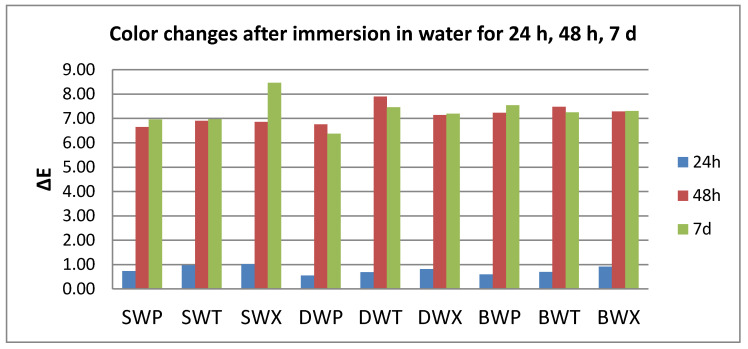
Mean values for ∆E parameter for all the tested values after immersion in distilled water.

C and W produced a significant color change amongst the tested samples after 24–48 h. For T and K immersion, significant differences were found both between 24 h and 48 h and between 48 h and 7 days (Figure 2, Figure 3, Figure 4 and Figure 5).

Related to the material, the influence of the beverages is insignificant during the whole time.

Related to the cleaning method (the chemical cleaning procedures) only after 24 h cleaning with P, the color change is significantly lower than after brushing. Otherwise, the cleaning method does not influence the color change.

Related to the mechanical cleaning procedure, X did not report significant changes.

### 3.2. Surface Roughness Measurements

Thermoforming does not significantly influence the mean roughness (*p* = 0.390). 

After 24 h immersion, all surface roughness values were below 0.25 µm. 

Related to the immersion beverages, roughness values increase significantly for coffee, tea, and water (T > C > W). Significant differences were also registered between T and K (*p* = 0.04).

Related to the material, S plates registered significantly higher roughness values than D. Related to the cleaning methods, both chemical and mechanical, roughness values were insignificantly different.

After 48 h immersion, surface roughness values were below 0.23 µm. Related to the immersion beverage, mean roughness values decreased in this order: T > C > K > W. Values increased significantly after immersion in all beverages (*p* < 0.05), but between the mediums, the differences remained insignificant. Regarding the material, differences were insignificant; roughness values decrease S > D > B. The cleaning methods also did not influence the mean roughness value (Ra = 0.12 µm) significantly.

After 7 days, the damage kept the order T > C > K > W Significant differences were between tea and water. The highest registered value was 0.28 µm. Related to the material, S plates showed significantly higher values than D and B (*p* = 0.046, respective, *p* = 0.0003), with mean values between 0.11 and 0.16 µm. Related to the cleaning method, both chemical and mechanical, the values are insignificant different (X > P > T).

**Figure 6:**
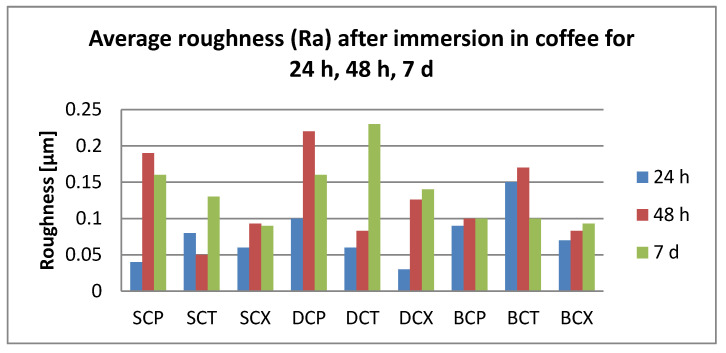
Mean values for average surface roughness (Ra) for the tested samples after immersion in coffee.

**Figure 7:**
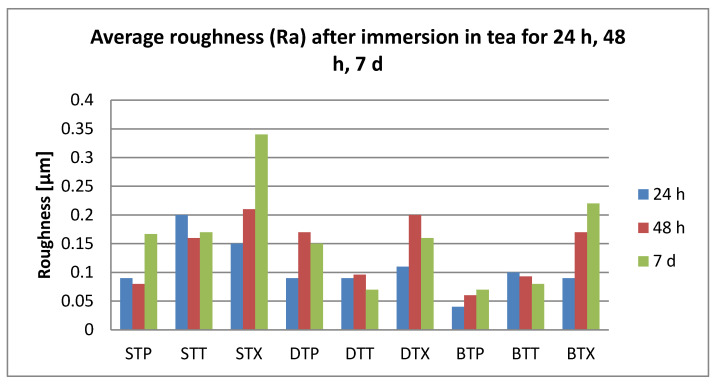
Mean values for average surface roughness (Ra) for the tested samples after immersion in tea.

**Figure 8:**
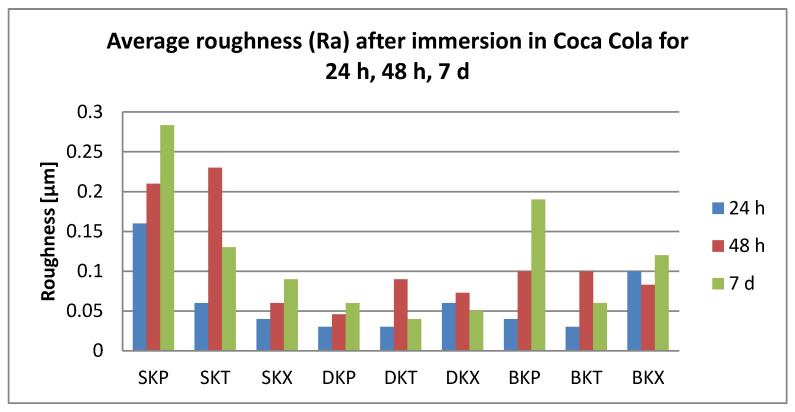
Mean values for average surface roughness (Ra) for the tested samples after immersion in Coca-Cola.

**Figure 9:**
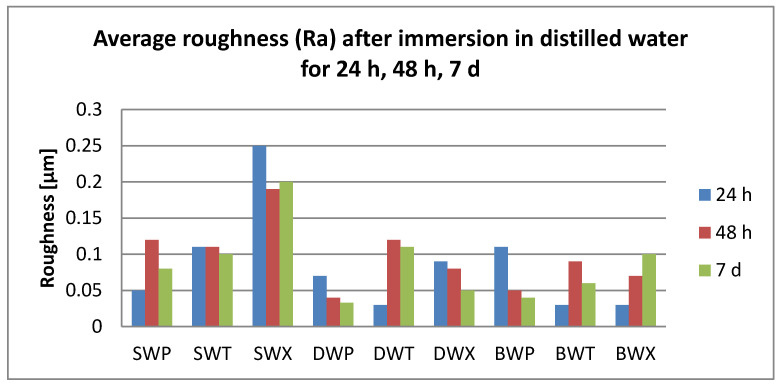
Mean values for average surface roughness (Ra) for the tested samples after immersion in distilled water.

The roughness values related to the immersion beverage are presented in Figure 6, Figure 7, Figure 8 and Figure 9.

Spearman correlation revealed a very weak relationship between microroughness and color change (0.133), after 7 days of immersion.

### 3.3. AFM and SEM Evaluations

AFM generated detailed 3D images of the surface topography. The images represent top views of the samples, offering information on the depth of the samples in the Z-direction, color coded. The light regions represent the peaks, and the dark regions represent the pores [18]. 

**Figure 10:**
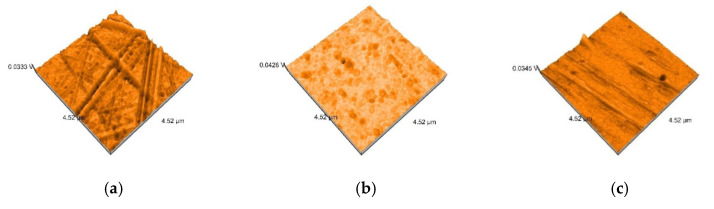
3D atomic force microscopy (AFM) images of the samples before thermoforming: (**a**) S; (**b**) D; (**c**) B.

**Figure 11:**
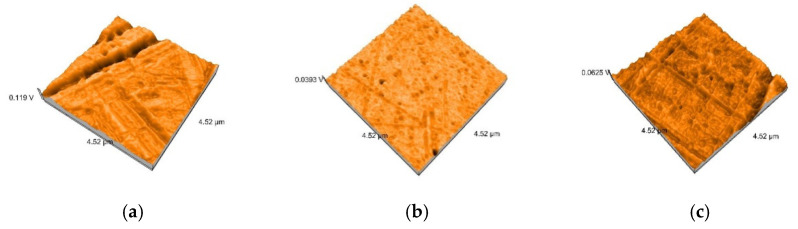
3D AFM images of the thermoformed samples: (**a**) S; (**b**) D; (**c**) B.

**Figure 12:**
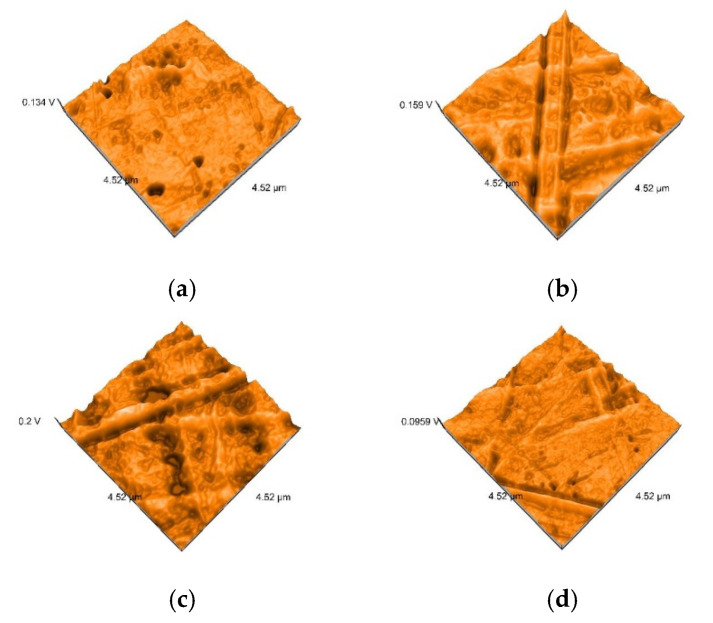
3D AFM images of the thermoformed S samples after mechanically brushing: (**a**) SCX (Sa = 7.5 nm, Sp = 52 nm, Sv = −43 nm); (**b**) SKX (Sa = 8.7 nm, Sp = 59 nm, Sv = −51 nm); (**c**) SWX (Sa = 22 nm, Sp = 85 nm, Sv = −66 nm); (**d**) STX (Sa = 43 nm, Sp = 89 nm, Sv = −98 nm).

**Figure 13:**
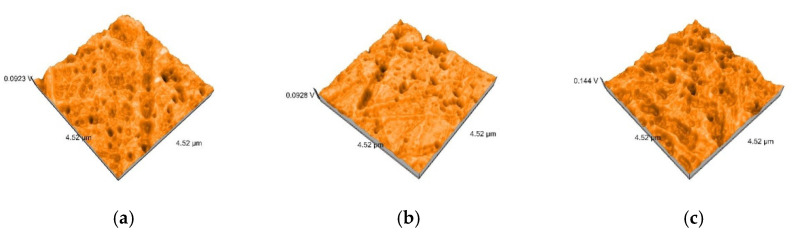
3D AFM images of the thermoformed D samples immersed in K: (**a**) DKP (Sa = 7 nm, Sp = 26 nm, Sv = −32 nm); (**b**) DKT (Sa = 6.7 nm, Sp = 58 nm, Sv = −27 nm); (**c**) DKX (Sa = 8.6 nm, Sp = 43 nm, Sv = −50 nm).

**Figure 14:**
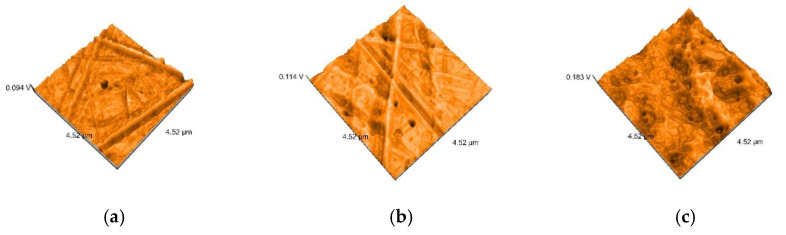
3D AFM images of the thermoformed B samples immersed in W: (**a**) DKP BWP (Sa = 23 nm, Sp = 52 nm, Sv = −56 nm); (**b**) BWT (Sa = 48 nm, Sp = 110 nm, Sv = −99 nm); (**c**) BWX (Sa = 15 nm, Sp = 63 nm, Sv = −54 nm).

AFM 3D images show non-uniform surfaces. S samples are characterized mainly by lineal projections, few pores, an entire irregular area, and narrow, deep scratch lines (Figure 10a). D samples demonstrated an irregular, more uniform surface, with rounded projections and pores (Figure 10b). Moderate and slight relief with heights and valleys, more prominent lines, were displayed on B samples (Figure 10c). The topography is more pronounced after thermoforming (Figure 11) and after de evaluation period using immersion beverages and cleaning methods (Figure 12, Figure 13 and Figure 14). 

Sa values before thermoforming are 1.2 nm for S, 1.8 nm for D, and 2 nm for B. After thermoforming, they increase to 6.5 nm, 7.1 nm, and respective 4.3 nm (Figure 10 and Figure 11).

After 7 days of immersing and periodical cleaning, Sa values range from 1.1 nm to 48 nm. Sp and Sv values reflect surface degradation. For S samples, the Sa, Sp, and Sv values are higher for the group, which was mechanically brushed (Figure 12). For D samples the nanoroughness was increased for the group immersed in K, followed by W (Figure 13), and for B samples for the group immersed in W, followed by K (Figure 8). 

AFM showed non-uniform surfaces, with distinct limited or sharp linear projections, and pores. Narrow, deep scratch lines cross the surfaces cause irregularities. All these demonstrated a moderate irregular surface with heights and valleys, with repercussions on the topography and oral behavior of the appliances.

**Figure 15:**
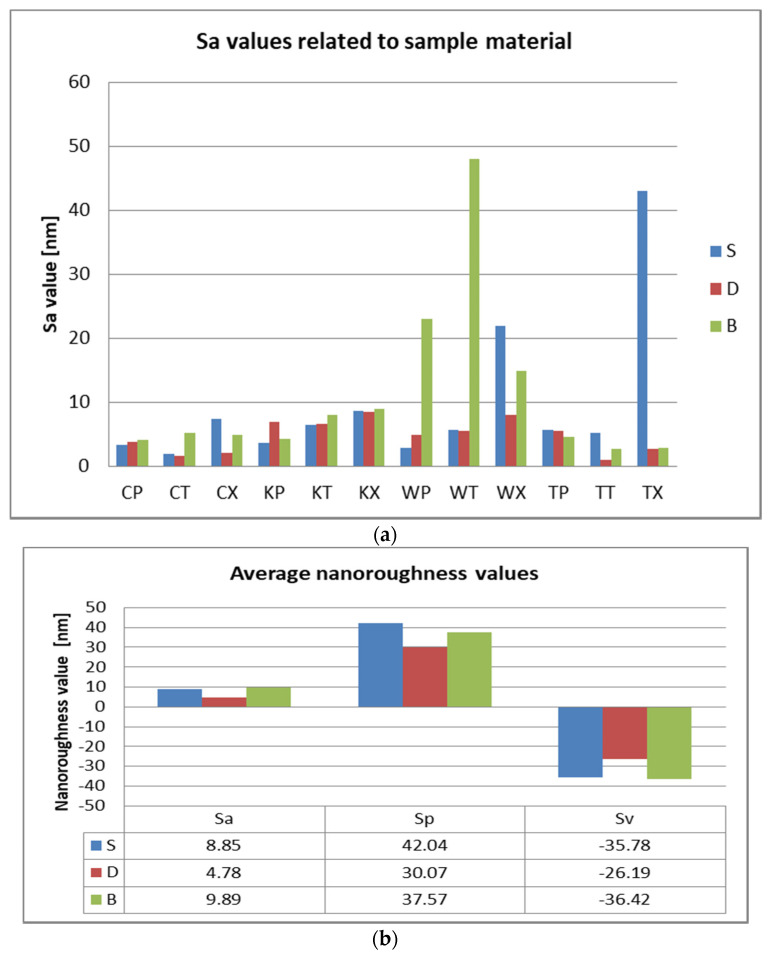
Nanoroughness values related to sample material (S, D, B): (**a**) Sa values. (**b**) average Sa, Sp, and Sv values.

Related to mean nanoroughness values Sa, D samples have demonstrated the most constant behavior, while B samples are visibly influenced by W (Figure 15a). This can be explained by the difference in the structure of the materials. Referring to mean values Sa, as well as to peak height values Sp, valley depth values Sv, D samples registered the lowest values, while the other two (S and B) intersect as values (Figure 15b).

**Figure 16:**
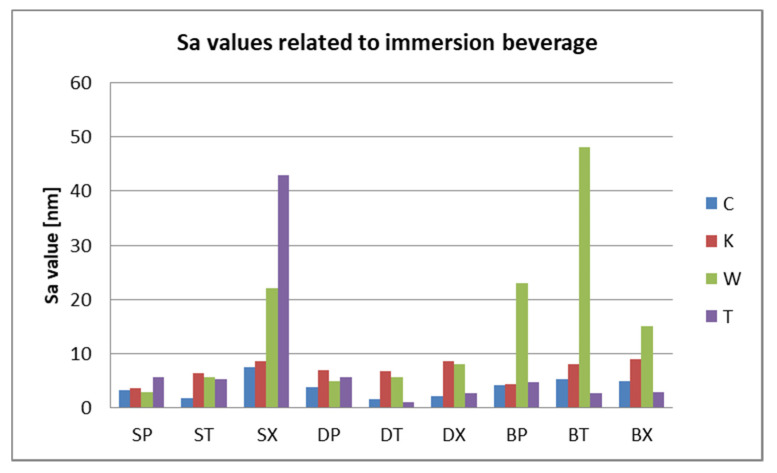
Sa values related to the immersion beverage.

**Figure 17:**
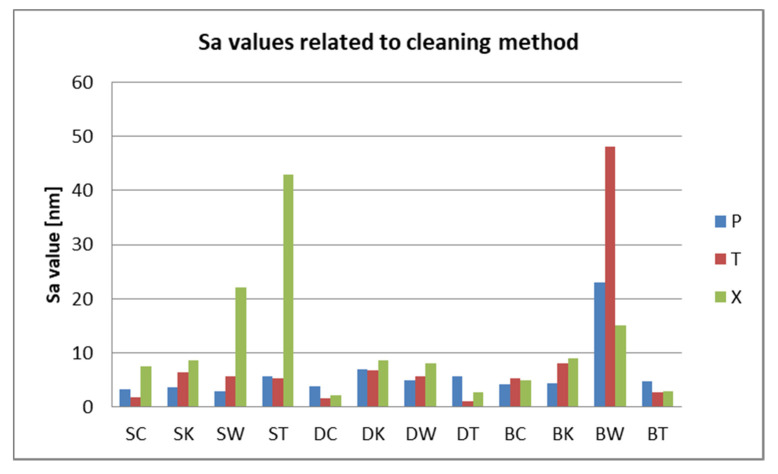
Sa values related to the cleaning method.

Related to immersion beverage and cleaning method, nanoroughness values support the 3D images, showing the influence of W on B samples and of the mechanical cleaning method on S samples (Figure 16 and Figure 17). This is explained by the difference between the structure of the studied materials. From the S samples, SX reported higher nanoroughness after immersion in tea and distilled water. Coffee influenced very little the nanoroughness.

In the D group, higher nanoroughness was reported for the Coca-Cola beverages, followed again by distilled water.

In the B group, significant changes were seen, especially after immersion in distilled water for the BT samples.

Brushing had a significant effect on all nanoroughness samples, but especially on ST and SW. Tablets affected most the BW samples.

**Figure 18:**
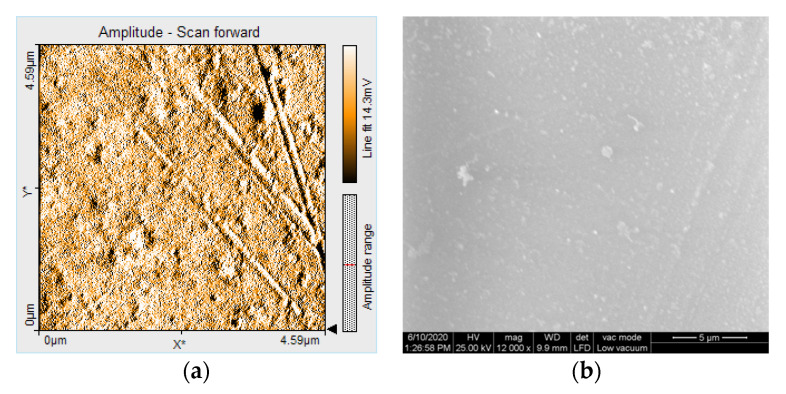
(**a**) Scanning amplitude (AFM) and (**b**) corresponding SEM image for a sample (DCT) with low nanoroughness values: Sa = 1.6 nm, Sp = 5.8 nm, Sv = −12 nm.

**Figure 19:**
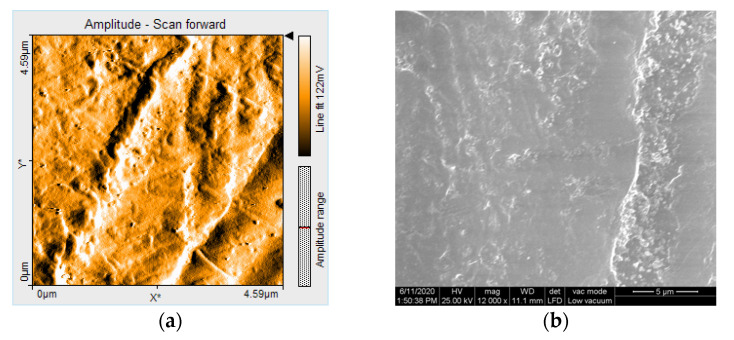
(**a**) Scanning amplitude (AFM) and (**b**) corresponding SEM image for a sample (BWX) with high nanoroughness values: Sa = 15 nm, Sp = 63 nm, Sv = −54 nm.

Under SEM evaluation, samples with lower nanoroughness values showed more homogeneous surface textures, and to those with higher nanoroughness values narrow scratches were found. Figure 18 and Figure 19 show the correlation of the scanning amplitude (AFM) and corresponding SEM image.

## 4. Discussion

The term surface quality refers to a set of widely different properties, such as color, roughness, and topography [19]. In the present study, the characteristics of color, roughness, morphology, and topography were selected to assess removable thermoplastic appliances surfaces. The surface quality is on the one hand material- and processing-related, and on the other hand subject to changes related to oral environment and cleaning methods. Polyethylene terephthalate glycol, commonly known as PETG or PET-G, is thermoplastic polyester with high chemical resistance, durability, and excellent formability for manufacturing. It can be easily thermoformed at low temperatures and is used in prosthetic and orthodontic splint therapy [20].

This kind of thermoformed materials benefit from a highly transparent appearance, but their color changes have been associated with pigment adsorption or penetration of the material surface after immersing in staining agents [10]. Variations in the color changes are attributed to the materials’ surface characteristics, such as roughness, which might accelerate pigment accumulation [16]. The study demonstrated that there is a very weak relationship between roughness and color modification. This could explain the color modification by the penetration of the material, not only by the effect on the surface.

Studies related that the translucency of thermoplastic plates decreases after thermoforming, due to the modification from amorphous to crystalline structures, related to the temperature, pressure, and working time [6,21]. These investigations revealed an insignificant color change after thermoforming.

Different studies suggest that the effects of temperature and water absorption on the properties of thermoplastic materials in a simulated oral environment are related to the materials [22]. Polymer materials absorb water from the air or immersion in water, and the morphological changes are related to hydrolytic degradation. PET-G sheets are known to be high water absorption polymers, with a hygroscopic expansion [5]. The investigations support this view, given that the changes are associated with both colored beverages and water, and they are not temperature-dependent. Hydrolysis is the process in which water reacts chemically with the polymer matrix, leading to several changes in their structure and their properties. The polymers are irreversibly degraded. Studies found that dimensional changes were reported to the studied PET-G materials. Water penetrates the areas of polymers and changes the structure and their surface. The water absorption process produces changes in polymer materials because water reacts as a spacer between chains. Studies demonstrated that water penetrates the amorphous regions of polymers and that crystalline regions remain unaffected by water at room temperature, whereas another study concluded that crystalline fractions of polymer were, in fact, also affected by water addition. All these changes affect the nanoroughness of the materials [23,24,25,26].

Studies described changes in the physical characteristics of PET-G after intraoral exposure and smoother surface and no crystallization of the PET-G sheets after thermoforming [23]. The results of this study show, on the contrary, an insignificant increase in roughness after thermoforming.

The assessment of the specimen surfaces under SEM after disinfection did not reveal noticeable damages in surface morphology [25].

Surface characteristics of dental materials, quantified in the roughness, are important because of the influence on bacterial adhesion and subsequent demineralization at the dental surface. Factors like material characteristics, exposure of these in the oral environment, and parameters of thermoforming, are found to contribute to the roughness of the PET-G materials [26,27,28]. It is known that the mean roughness (Ra) of dental materials should be below 0.2 µm in order to prevent the accumulation of plaque and microorganisms [29,30,31]. An increase in surface roughness leads to an increase plaque accumulation over time [32]. Studies state that an intraoral hard surface can cause discomfort and can be detected by the tongue if the roughness value exceeds 0.5 µm [33]. The mean roughness values of the investigated materials do not exceed 0.25 µm irrespective of immersion beverage, time, temperature, and cleaning method.

The qualitative examination of the surface topography allowed the evaluation of the inherent roughness of the material and the destructive effect of the beverages and cleaning methods. However, SEM has limitations related to surface topography description because it does not allow the visualization of 3D surface texture. AFM can also be used for measurements and 3D evaluations and a more detailed description of the surface topography [24].

Roughness values recorded by profilometers allow a quantitative measure of the surface irregularities, but Ra parameter is considered as a poor indicator of surface texture, even it is most frequently used to evaluate surface topography in dental materials [24,34,35,36,37,38,39,40,41,42]. Surface topography is 3D in nature, and therefore parameters obtained from 3D AFM evaluations are more realistic. Compared to the 5 μm diamond stylus of the profilometer, the AFM is equipped with a 0.01 μm tip, which permits more precise measurements.

The tiny peaks are responsible for the improved roughness [Adegbola]. AFM allows the investigation of such materials with low hardness. It allows a precise measurement of surface quality, providing high dimensional accuracy. It is very important to examine the roughness at the nanolevel [42,43,44].

The water adsorption of the polymer matrix during immersion causes hydrolytic degradation, resulting in increased Ra [26,37]. Th investigations to confirm this aspect.

Proper maintenance of the properties of the removable appliances in the oral environment will continue to be a critical aspect [12]. This study highlighted the color changes, which become extremely marked after 48 h of immersion, no matter that the liquid is colored or no, and regardless of temperature. The limitations of this study come from a small number of materials that were studied and cleaning methods. Further studies have to be conducted to include more materials and more cleaning methods.

## 5. Conclusions

Within the limitations of this study, the following conclusions can be drawn:

1. Quantitative color change evaluations revealed a slight change in color after 24 h and an extremely marked change after 48 h, respective 7 days, for all tested specimens, regardless of the type of material, beverage, or cleaning method.
2. Related to the microroughness, values are below the clinically acceptable limit of 0.20 µm for all samples, all the time during the investigations.
3. Related to the type of material, color changes are insignificant, and roughness values registered some significant variations, but with the preservation of the values below the accepted limit.
4. Related to mean nanoroughness values Sa, and 3D evaluations of the surface quality, D samples have demonstrated the most constant behavior, while B samples are svisibly influenced by W. Related to the cleaning method, nanoroughness values support the 3D images, showing the influence of the mechanical cleaning method on S samples.

## Figures and Tables

**Table 1:** Heating and cooling time used during the thermoforming process.

Material	Pressure (Bar)	Heating Time (Seconds)	Cooling Time (Seconds)
Duran (Scheu-Dental GmbH, Iserlohn, Germany)	4.7	30	60
Biolon (Dreve Dentamid GmbH, Unna, Germany)	4.7	40	50
Crystal (Bio Art Dental Equipment, Sao Carlos, Brazil)	4.7	30	60

**Table 2:** Abbreviations used for the samples in the study.

No.	Material/Beverage/Cleaning Method		Abbreviation
1.	material	Duran (Scheu-Dental GmbH, Iserlohn, Germany)	S
		Biolon (Dreve Dentamid GmbH, Unna, Germany)	D
		Crystal (Bio Art Dental Equipment, Sao Carlos, Brazil)	B
2.	beverage	Coffee	C
		Tea	T
		Coca-cola	K
		distilled water	W
3.	cleaning method	Centron Cleaning Powder (Scheu, Iserlohn, Germany)	P
		Corega Cleanser Tablets (Stafford-Miller, Dungarvan, Ireland)	T
		Brushing	X

**Table 3:** Levels of color change, according to the national bureau of standards (NBS).

NBS Units	Color Changes
0.0–0.5	extremely slight change
0.5–1.5	slight change
1.5–3.0	perceivable
3.0–6.0	marked change
6.0–12.0	extremely marked change
12.0 or more	change to another color
